# Meta-Analysis of *in vitro*-Differentiated Macrophages Identifies Transcriptomic Signatures That Classify Disease Macrophages *in vivo*

**DOI:** 10.3389/fimmu.2019.02887

**Published:** 2019-12-11

**Authors:** Hung-Jen Chen, Andrew Y. F. Li Yim, Guillermo R. Griffith, Wouter J. de Jonge, Marcel M. A. M. Mannens, Enrico Ferrero, Peter Henneman, Menno P. J. de Winther

**Affiliations:** ^1^Department of Medical Biochemistry, Experimental Vascular Biology, Amsterdam University Medical Centers, University of Amsterdam, Amsterdam, Netherlands; ^2^Genome Diagnostics Laboratory, Department of Clinical Genetics, Amsterdam University Medical Centers, University of Amsterdam, Amsterdam, Netherlands; ^3^Epigenetics Discovery Performance Unit, GlaxoSmithKline, Stevenage, United Kingdom; ^4^Tytgat Institute for Liver and Intestinal Research, Amsterdam University Medical Centers, University of Amsterdam, Amsterdam, Netherlands; ^5^Computational Biology, Target Sciences, GlaxoSmithKline, Stevenage, United Kingdom; ^6^Institute for Cardiovascular Prevention (IPEK), Ludwig Maximilians University, Munich, Germany

**Keywords:** meta-analysis, elastic net classification, macrophages, alveolar macrophages (AMs), synovial macrophages (SMs), adipose tissue macrophages (ATMs), macrophage identifier (macIDR)

## Abstract

Macrophages are heterogeneous leukocytes regulated in a tissue- and disease-specific context. While *in vitro* macrophage models have been used to study diseases empirically, a systematic analysis of the transcriptome thereof is lacking. Here, we acquired gene expression data from eight commonly-used *in vitro* macrophage models to perform a meta-analysis. Specifically, we obtained gene expression data from unstimulated macrophages (M0) and macrophages stimulated with lipopolysaccharides (LPS) for 2–4 h (M-LPS_early_), LPS for 24 h (M-LPS_late_), LPS and interferon-γ (M-LPS+IFNγ), IFNγ (M-IFNγ), interleukin-4 (M-IL4), interleukin-10 (M-IL10), and dexamethasone (M-dex). Our meta-analysis identified consistently differentially expressed genes that have been implicated in inflammatory and metabolic processes. In addition, we built macIDR, a robust classifier capable of distinguishing macrophage activation states with high accuracy (>0.95). We classified *in vivo* macrophages with macIDR to define their tissue- and disease-specific characteristics. We demonstrate that alveolar macrophages display high resemblance to IL10 activation, but show a drop in IFNγ signature in chronic obstructive pulmonary disease patients. Adipose tissue-derived macrophages were classified as unstimulated macrophages, but acquired LPS-activation features in diabetic-obese patients. Rheumatoid arthritis synovial macrophages exhibit characteristics of IL10- or IFNγ-stimulation. Altogether, we defined consensus transcriptional profiles for the eight *in vitro* macrophage activation states, built a classification model, and demonstrated the utility of the latter for *in vivo* macrophages.

## Introduction

The spatiotemporal regulation of tissue homeostasis relies on the complex network of diverse and heterogeneous immune cell populations. As a highly plastic and multifunctional immune cell, macrophages play a decisive role in the balance between pro-inflammatory defense and anti-inflammatory tissue repair (1). Dysregulation of macrophages has been implicated in a variety of disorders. As *in vivo* macrophages are often difficult to obtain in sufficient quantities, peripheral blood monocyte derived macrophages (MDMs) have been used extensively as *in vitro* model systems. To mimic *in vivo* macrophages encountering various triggers, MDMs are stimulated *in vitro* with lipopolysaccharides (LPS) and/or interferon-γ (IFNγ) to generate pro-inflammatory macrophages (M1), or activated with interleukin-4 (IL4), interleukin-10 (IL10) or glucocorticoids to generate anti-inflammatory macrophages (M2) (2). While *in vitro* model systems based on differential activation of MDMs emerge as a practical heuristic, they are not identical to *in vivo* tissue resident macrophages or infiltrating MDMs, which are often shaped by a complex and dynamic milieu within the microenvironment (1). Nonetheless, within the fast-growing field of systems immunology a crucial need exists for identifying and defining *in vivo* macrophage populations, as well as identifying an *in vitro* model capable of mimicking the tissue physiology and the systemic perturbation associated with diseases (3).

In the most comprehensive expression profiling study to date on *in vitro* macrophages, Xue et al. showed that macrophages display a more divergent reprogramming, thereby extending the classical pro- and anti-inflammatory dichotomy to an activation spectrum (4). While there have been attempts to summarize published studies in an effort to attain consensus for *in vitro* models (5), a proper integrative analysis on multiple published datasets has thus far not been performed. To address this, we integrated 206 microarray and bulk RNA-sequencing (RNA-seq) datasets from 19 different studies (6–23) to systematically characterize eight *in vitro* MDM activation states. Specifically, we investigated unstimulated macrophages (M0) and macrophages activated by: short exposure (2–4 h) to LPS (M-LPS_early_) or long exposure (18–24 h) to LPS (M-LPS_late_), LPS with IFNγ (M-LPS+IFNγ), IFNγ (M-IFNγ), IL-4 (M-IL4), IL-10 (M-IL10), and dexamethasone (M-dex) (Table 1). First, we identified consistently differentially expressed genes (cDEGs) and the associated pathways by comparing activated with unstimulated macrophages using a random effects meta-analysis (36). Second, we implemented penalized multinomial logistic regression and trained a classifier through repeated cross-validation that was capable of accurately and robustly distinguishing MDM activation states, independent of the macrophage differentiation factor applied and transcriptomic platform used. We named this classification model macIDR (macrophage identifier) and used it to project *in vivo* tissue-isolated and disease-associated macrophages onto the eight *in vitro* MDMs (26–29, 31–35) in an effort to identify potential macrophage-associated differences when comparing investigating tissues from different patient groups.

**Table 1:** Included datasets.

**References**	**Type**	**Dataset ID**	**Purpose**	**Isolation method for monocyte/macrophage**
Fuentes-Duculan et al. (6)	Microarray	GSE18686	Training & test	Adherent PBMCs
Schroder et al. (7)	Microarray	GSE19765	Training & test	CD14+ MACS microbead selection
Benoit et al. (16)	Microarray	GSE30177	Training & test	FACS verified after differentiation
Chandriani et al. (17)	Microarray	GSE47538	Training & test	CD14+ MACS microbead selection
Martinez et al. (18)^*^	Microarray	GSE5099	Training & test	CD14+ MACS microbead selection
Derlindati et al. (19)	Microarray	GSE57614	Training & test	Dynabeads negative isolation, FACS verified
Jubb et al. (20)	Microarray	GSE61880	Training & test	CD14+ MACS microbead selection
Steiger et al. (21)	Microarray	GSE79077	Training & test	CD14+ MACS microbead selection
Fujiwara et al. (22)	Microarray	GSE85346	Training & test	CD14+ MACS microbead selection
Tsang et al. (8)	Microarray	E-MEXP-2032	Training & test	Adherent PBMCs
Przybyl et al. (9)	Microarray	E-MTAB-3309	Training & test	Adherent PBMCs
Byng-Maddick et al. (10)	Microarray	E-MTAB-5095	Training & test	Adherent PBMCs
Surdziel et al. (11)	Microarray	E-MTAB-5913	Training & test	CD14+ MACS microbead selection
	RNAseq	BLUEPRINT	Training & test	CD14+ MACS microbead selection
Park et al. (12)	RNAseq	GSE100382	Training & test	CD14+ MACS microbead selection
Zhang et al. (13)	RNAseq	GSE55536	Training & test	Adherent PBMCs
Martins et al. (14)	RNAseq	GSE80727	Training & test	Dynabeads negative isolation
Realegeno et al. (15)	RNAseq	GSE82227	Training & test	CD14+ MACS microbead selection
Own	RNAseq	E-MTAB-7572	Test	CD14+ MACS microbead selection
Riera-Borull et al. (23)	Microarray	GSE99056	GM-CSF verification	CD14+ MACS microbead selection
Vento-Tormo et al. (24)	Microarray	GSE75938	GM-CSF verification, non-mac verification	CD14+ MACS microbead selection
Xue et al. (4)	Microarray	GSE46903	GM-CSF verification, non-mac verification	CD14+ MACS microbead selection
Tasaki et al. (25)	Microarray	GSE93776	Non-mac verification	FACS sorting
Yarilina et al. (26)	Microarray	GSE10500	Synovial macrophage test	CD14+ MACS microbead selection
You et al. (27)	Microarray	GSE49604	Non-mac verification, synovial macrophage test	CD14+ MACS microbead selection
Kang et al. (28)	Microarray	GSE97779	Synovial macrophage test	CD14+ MACS microbead selection
Asquith et al. (29)	Microarray	E-MEXP-3890	Synovial macrophage test	CD14+ MACS microbead selection
Stephenson et al. (30)	scRNAseq	phs001529.v1.p1	Synovial macrophage test	Single cell RNA-seq
Shaykhiev et al. (31)	Microarray	GSE13896	Alveolar macrophage test	Adherent bronchoalveolar lavage cells, Diff-Quik staining verified
Woodruff et al. (32)	Microarray	GSE2125	Alveolar macrophage test	Adherent bronchoalveolar lavage cells, Diff-Quik staining verified
Madore et al. (33)	Microarray	GSE22528	Alveolar macrophage test	Adherent bronchoalveolar lavage cells, Diff-Quik staining verified
Goleva et al. (34)	Microarray	GSE7368	Alveolar macrophage test	Filtered bronchoalveolar lavage cells
Dalmas et al. (35)	Microarray	GSE54350	Adipose tissue macrophage test	Positive selection magnetic beads

An overview of the datasets and the associated studies included in the in the meta-analysis and the classification analysis.

* *The GSE5099 dataset was composed of two Affymetrix microarray datasets: U133A and U133B. Due to the limited overlap in genes between U133B with the rest, we only included the U133A dataset*.

## Methods

### Data Sources and Search Strategy

**Figure 1:**
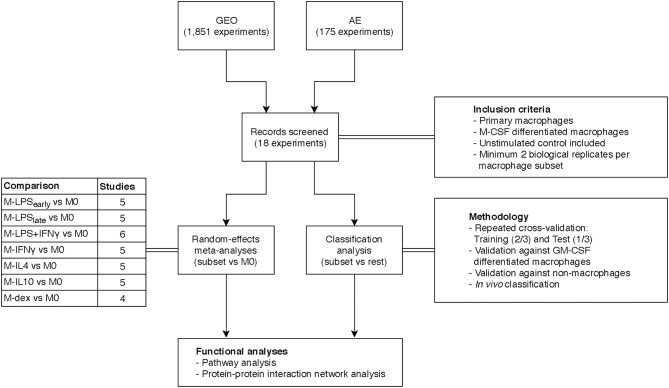
Overview study design. Transcriptome datasets were found on the Gene Expression Omnibus (GEO) or ArrayExpress (AE) and screened according to the inclusion criteria yielding 18 datasets. A separate meta-analysis and classification analysis was performed and results thereof were subjected to functional pathway analyses.

Our initial search consisted of identifying public datasets for the meta-analysis and classification analysis, which we called the “training and test” data. Specifically, we searched the National Center for Biotechnology Information (NCBI) Gene Expression Omnibus (GEO) (37) and European Bioinformatics Institute (EBI) ArrayExpress (AE) (38) using the keywords “(macrophage) OR (monocyte) OR (MDM) OR (HBDM) OR (MoDM) OR (MAC) OR (dendritic cell) AND “Homo sapiens” [porgn:__txid9606].” Our search yielded 1,851 and 175 experiments for GEO and AE, respectively at the time of writing (May 2018). In a subsequent screening we filtered for studies that investigated primary macrophages where we excluded studies that investigated stem cell derived macrophages or immortalized cell lines. For consistency purposes, we limited the training and test data to macrophages differentiated with macrophage colony-stimulating factor (M-CSF) only as it is the most commonly used method to generate MDMs for both pro- and anti-inflammatory activations. We then categorized macrophage activation states according to stimulus and treatment time. For the meta-analysis and classification analysis, we sought to obtain at least 4 studies that included at least 2 biological replicates per activation state. Furthermore, studies were selected only if they included unstimulated control macrophages. Microarray datasets generated on platforms other than Illumina, Affymetrix or Agilent were excluded to ensure comparability. As we only investigated genes that were measured in every single study, datasets that displayed limited overlap in the measured genes with the other studies were also removed. To further validate the classification model, we expanded our search by acquiring data from granulocyte-macrophage colony-stimulating factor (GM-CSF) differentiated macrophages as well as data from non-macrophage cells. Finally, we expanded our search to include data from *in vivo* macrophages obtained from clinical specimen. An overview of our inclusion criteria and the general workflow can be found in Figure 1.

### Data Extraction and Import

#### Microarray Data Preparation

All analyses were performed in the R statistical environment (v3.5.0) (39). The download and import of the raw and processed GEO and AE microarray was performed using the GEOquery (v2.48.0) (40) and the ArrayExpress (v1.40.0) (41) packages respectively. Raw data was normalized in a platform-specific fashion: Affymetrix microarrays were normalized using the rma function from the affy (v1.54.0) (42) and oligo (v1.40.2) (43) packages, whereas Illumina and Agilent microarrays were normalized using the neqc function from the limma (v3.31.14) (44) package. Quality control of the log_2_ transformed expression values was performed using WGCNA (v1.51) (45) and arrayQualityMetrics (v3.32.0) (46) to remove unmeasured samples, genes, and studies of insufficient quality. Microarray probes were reannotated to the Entrez ID according to the annotation files on Bioconductor. Probes that associated to multiple Entrez IDs were removed and multiple probes associating to the same Entrez ID were summarized by taking the median.

#### Bulk RNA Sequencing Data Preparation

Public raw sequencing reads were sourced from the NCBI Sequence Read Archive (SRA) (47) and converted to fastq files using the fastq-dump function from the SRA-tools package (v2.9.0). All raw fastq files were first checked for quality using FastQC (v0.11.7) (48) and MultiQC (v1.4) (49). The sequencing reads were aligned against the human genome GRCh38 using STAR (v2.5.4) (50). Post-alignment processing was performed using SAMtools (v1.7) (51) after which reads overlapping gene features obtained from Ensembl (v91) were counted using the featureCounts program in the Subread (v1.6.1) (52) package. Gene annotations were converted from Ensembl IDs to Entrez IDs using biomaRt (53).

#### Single-Cell RNA Sequencing Data Preparation

Processed count data was downloaded and analyzed in the R statistical environment. Normalization, scaling, clustering was done using the Seurat package (v3.0.2) (54). Macrophages were identified based on unsupervised Louvain clustering and identifying the cluster that expressed typical macrophage markers *SPI1, C1QB, CD14, CD68*, and *CSF1R*. The purported macrophage cells were extracted and a secondary unsupervised clustering analysis was performed. Classification of the individual was subsequently performed without the use of an intercept.

### Data Analysis

#### Meta-Analysis

A random effects meta-analysis was performed on the normalized data using the GeneMeta package (v1.52.0) (55), which implements the statistical framework outlined by Choi et al. (36). In short, the standardized effect size (Cohen d adjusted using Hedges and Olkin's bias factor) and the associated variance were calculated for each study by comparing each activated macrophage with the unstimulated macrophage within each study. The standardized effect sizes were then compared across studies by means of random effects model to correct for the inter-study variation, thereby yielding a weighted least squares estimator of the effect size and its associated variance. The estimator of the effect size was then used to calculate the Z-statistic and the *p*-value, which was corrected for multiple testing using the Benjamini-Hochberg procedure. We modified the GeneMeta functions to incorporate the shrunken sample variances obtained from limma (44) for calculating the standardized effect sizes. Consistently differentially expressed genes (cDEGs) were defined by an adjusted *p* < 0.05.

#### Classification Analysis

Raw microarray and RNA-seq data were log_2_ transformed where necessary after which the data was (inner) merged and randomly divided into a training (2/3) and test set (1/3). As the test set should remain hidden from the training set, raw microarray and RNA-seq data was used instead of normalized data to prevent data leakage (56). Elastic net regression was subsequently performed using the R glmnet (v2.0) (57) package where we set the alpha to 0.8.

The training set was subjected to 10-fold cross-validation for tuning the regularization parameter lambda. This procedure was subsequently repeated 500 times to stabilize the randomness introduced by the initial splitting step for cross-validation (58). We considered genes to be stable classifiers if they displayed a non-zero log odds ratio in at least 50% of the 500 iterations. The final log odds ratio was selected by taking the median of each stable predictor gene across the 500 iterations. We subsequently validated our classification model on the withheld test set.

In addition to the training and test data, we downloaded and imported additional datasets from GM-MDMs, non-macrophage cells, and *in vivo* macrophages. Subsequent classification was performed using the macIDR package. Unlike the studies included for training and testing, some of the included studies were performed on platforms that did not measure the expression of some of the predictor genes. To that end, the relative log odds ratios were calculated, which represent the log odds ratio present relative to the total log odds ratio had all predictor genes been present.

#### Functional Gene Set Analyses

Gene set analyses of the 8000 cDEGs with the lowest *p*-values were performed using the Ingenuity Pathway Analysis (59) software package, whereas the predictor genes were functionally assessed using the Metascape software (60). Additionally, potential relationships between the cDEGs were assessed and visualized by performing a protein-protein association (PPA) analysis on the 100 cDEGs with the lowest *p*-values for each comparison using the STRING database (61). In short, the cDEGs were interrogated for cataloged associations with evidence from the literature that the encoded proteins interacted, co-expressed, or co-evolved with one another based on text mining, curated databases, and experiments. We limited the PPA-analyses to the top 100 cDEGs to make networks comparable across the different stimuli and to prevent overcrowding.

### *In vitro* Validation Experiment

#### Human Monocyte-Derived Macrophage Differentiation and Stimulation

Buffy coats from three healthy anonymous donors were acquired from the Sanquin blood bank in Amsterdam, the Netherlands. All the subjects provided written informed consent prior to donation to Sanquin. Monocytes were isolated through density centrifugation using Lymphoprep™ (Axis-Shield) followed by human CD14 magnetic beads purification with the MACS® cell separation columns (Miltenyi). The resulting monocytes were seeded on 24-well tissue culture plates at a density of 0.8 million cells/well. Cells were subsequently differentiated to macrophages for 6 days in the presence of 50ng/mL human M-CSF (Miltenyi) with Iscove's Modified Dulbecco's Medium (IMDM) containing 10% heat-inactivated fetal bovine serum, 1% Penicillin/Streptomycin solution (Gibco) and 1% L-glutamine solution (Gibco). The medium was renewed on the third day. After differentiation, the medium was replaced by culture medium without M-CSF and supplemented with the following stimuli: nothing, 10 ng/mL LPS (Sigma, E. coli E55:O5), 10 ng/mL LPS plus 50 ng/mL IFNγ (R&D), 50ng/mL IFNγ, 50 ng/mL IL4 (PeproTech), 50 ng/mL IL10 (R&D), 100 nM dexamethasone (Sigma) for 24 h. LPS_early_ macrophages were first cultured with culture medium for 21 h and then stimulated with 10 ng/mL LPS for 3 h prior to harvest. Total RNA was isolated with Qiagen RNeasy Mini Kit per the manufacturer's recommended protocol. RNA sequencing libraries were prepared using the standard protocols of NuGEN Ovation RNA-Seq System V2 kit. Size-selected cDNA library samples were sequenced on a HiSeq 4000 sequencer (Illumina) to a depth of 16M per sample according to the 50 bp single-end protocol at the Amsterdam University Medical Centers, location Vrije Universiteit medical center. Raw fastq files were subsequently processed in the same manner as the public datasets to maintain consistency.

## Results

### Overview Datasets

Altogether, we identified 206 suitable microarray and bulk RNA-sequencing (RNA-seq) datasets from 19 different studies (6–23) for the meta-analysis and classification analysis (Figure 1). These datasets comprised eight *in vitro* MDM activation states, namely unstimulated macrophages (M0) and macrophages activated by: short exposure (2 to 4 h) to LPS (M-LPS_early_) or long exposure (18 to 24 h) to LPS (M-LPS_late_), LPS with IFNγ (M-LPS+IFNγ), IFNγ (M-IFNγ), IL-4 (M-IL4), IL-10 (M-IL10), and dexamethasone (M-dex) (Table 1).

### Identification of Well-Known and Novel Transcriptional Markers and Pathways of Macrophage Activation

We identified genes that were consistently differentially expressed across studies (cDEGs) by means of a random effects meta-analysis (Supplementary Table 1). Many of the observed cDEGs were known to be characteristic for certain macrophage activation states. For example, interleukin-1 beta (*IL1B*), C-C Motif Chemokine Ligand 17 (*CCL17*) and Cluster of Differentiation 163 (*CD163*) were consistently upregulated in M-LPS_late_, M-IL4, and M-dex compared with M0, respectively (5). Notably, we identified several novel genes that were not widely considered as activated macrophage markers. Consistent upregulation of interleukin-7 receptor (*IL7R*) and CD163 Molecule Like 1 (*CD163L1*) was observed in M-LPS_early_ and M-IL10, respectively. Similarly, consistent downregulation of Nephroblastoma Overexpressed (NOV) and Adenosine A2b receptor (*ADORA2B*) was observed when comparing M-IFNγ, M-LPS_late_, and M-LPS+IFNγ with M0, respectively.

**Figure 2:**
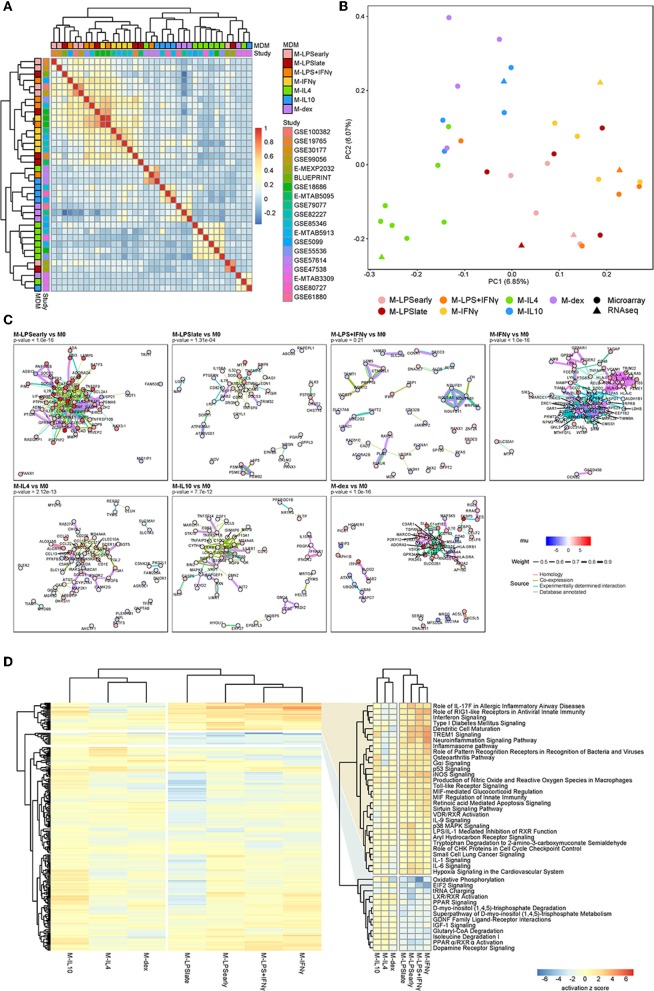
Summary meta-analysis. **(A)** Heatmap of the Cohen d pairwise Spearman correlation coefficients. **(B)** Principal component analysis of the Z-values obtained from the meta-analysis. **(C)** Protein-protein association network as obtained from the top 100 cDEGs using the STRING database. Node colors represent the unbiased estimator of the effect size (mu), whereas the edge colors and thickness represent the source of the cataloged association and the weight of the evidence. **(D)** Heatmap of the canonical pathways with the intensity representing the activation z score. Two most defining clusters have been enlarged and annotated on the right.

At the genome-wide level, pairwise correlation analysis of the standardized effect sizes across studies showed that M-LPS_early_, M-LPS_late_, M-IFNγ, and M-LPS+IFNγ formed one cluster, whereas M-IL4, M-IL10 and M-dex formed a second cluster (Figure 2A). Other activation states could not be discerned easily, which could be attributed to study-specific effects. This observation agrees with previous studies where transcriptional alterations induced with these conventional pro- and anti-inflammatory stimuli were found to cluster according to the M1-M2 dichotomy (4, 62). Further sub-clustering appeared to divide the macrophages according to the stimuli. The separation between the M1 and the M2 was also apparent as the first principal component (PC1), which displayed a clear separation between M1 and M2 associated stimuli on the left and right, respectively (Figure 2B). Relative to the M1 macrophages, the M2 macrophages appeared to display a stronger diversity along PC2, suggesting a more distinctive transcriptional programming of the individual activation states.

#### cDEGs Are Enriched for Pro- and Anti-inflammatory Pathways

To investigate the functional relevance of the cDEGs, we performed protein-protein association (PPA) analyses on the top 100 cDEGs. Per comparison, we observed cluster formation visually as well as statistically, where an overrepresentation analysis identified more PPAs than would have been observed by chance for a random gene set of similar size. Notably, M-LPS_early_, M-LPS_late_, M-IFNγ, M-IL4, M-IL10, and M-dex cDEGs all displayed significantly more interactions (Figure 2C). M-LPS_early_ cDEGs formed an upregulated PPA-network centered around the pro-inflammatory mediators tumor necrosis factor α (TNFα), interleukin-6 (IL6), and prostaglandin-endoperoxide synthase 2 (PTGS2). M-IFNγ cDEGs revealed two separate clusters, one for major histocompatibility complex (MHC) proteins (upregulated) and one for nucleic acid and nitrogen metabolism-associated proteins (downregulated). The PPA network found for M-IL4 cDEGs suppressed pro-inflammatory proteins interleukin-1B (IL1B) and interleukin-1 receptor-associated kinase 3 (IRAK3), but enhanced chemokine ligands (CCL) considered to be co-expressed with IL1B, such as CCL13, CCL17, CCL18, and CCL22 (Figure 2C). Finally, M-dex cDEGs displayed a cluster centered at V-Set And Immunoglobulin Domain Containing 4 (VSIG4) with many of the genes encoding anti-inflammatory proteins, such as Macrophage Receptor With Collagenous Structure (MARCO) (63) and membrane-spanning, four domain family, subfamily A (MS4A4A) (64). As VSIG4 has been shown to modulate macrophage inflammatory function via mitochondrial reprogramming (65), the PPA network in M-dex vs. M0 suggests concordant immunosuppressive metabolic changes in macrophages.

To systematically identify whether the cDEGs were enriched for particular pathways, we performed pathways analyses using the Ingenuity Pathway Analysis (IPA) software package. While the clusters were slightly different among individual macrophage activation states, the overall separation between M1 and M2 was apparent with hierarchical clustering revealing two sets of pathways that appeared to be responsible for the separation (Figure 2D and Supplementary Table 2). Pathways known for their pro- and anti-inflammatory responses, such as interferon signaling and LXR/RXR activation, displayed a clear and distinct pattern for the M1 and M2 macrophages, respectively.

### Elastic Net Classification

#### Classification Analysis Distinguishes Macrophage Activation States With High Accuracy

**Figure 3:**
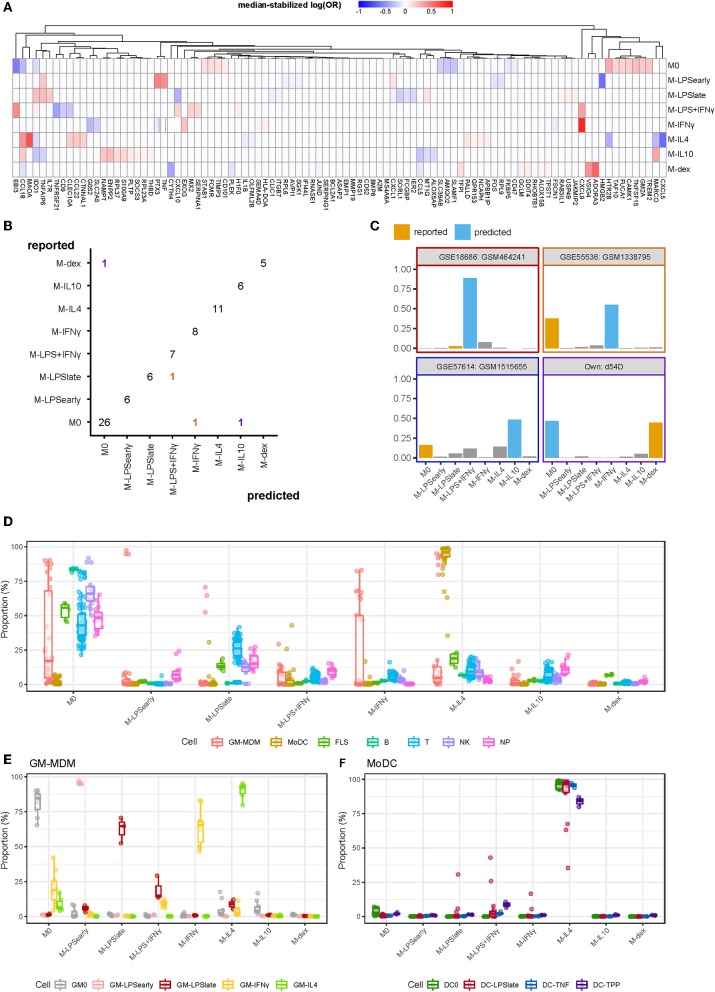
Summary classification analysis. **(A)** Heatmap of the median-stabilized log odds ratios per macrophage activation state for each of the 97 predictor genes. **(B)** Confusion matrix representing the number of correctly classified samples (entries on the diagonal) vs. the misclassified samples (entries on the off-diagonal). Classes on the y-axis represents the reported class while classes on the x-axis represent the predicted class. Colors represent the predicted classes with purple, red, yellow, and blue representing M-dex, M-LPS+IFNγ, M-IFNγ, and M-IL10, respectively. **(C)** Bar plots of the misclassified samples depicting the classification signal on a scale of 0 to 1 where the class with the largest signal represents the predicted class. Blue bars represent the incorrectly predicted class and orange bars represent the reported class. Border colors represent the predicted classes with purple, red, yellow, and blue representing M-dex, M-LPS+IFNγ, M-IFNγ, and M-IL10, respectively. **(D)** Boxplots representing the classification signal on a scale of 0 to 1 where classes with the largest signal represents the predictions. Colors represent GM-CSF differentiated macrophages (GM-MDMs), monocyte-derived dendritic cells (MoDCs), fibroblast-like synoviocytes (FLS), B lymphocytes (B), T lymphocytes (T), natural killer cells (NK), and neutrophils (NP). **(E)** GM-MDMs and **(F)** MoDCs colored by stimulation.

We merged the data from different microarray and RNA-seq experiments and performed multinomial elastic net regression on the 5986 overlapping genes present in all datasets to identify gene sets capable of distinguishing macrophage activation states. The merged expression data was randomly split into a training (2/3) and a test (1/3) set whereupon the training set was used to build a model through 10-fold cross-validation. To achieve robust performance, we repeated the cross-validation procedure 500 times and took the median thereof to stabilize the log odds ratios (58). Genes were considered stable predictors if their log odds ratio was non-zero in more than 50% of the iterations (Supplementary Figure 1). Subsequent classification was performed using the median log odds ratio per gene across all iterations (Figure 3A). Altogether, our classifier was composed of 97 median-stabilized predictor genes, and was compiled as an R package called macIDR (https://github.com/ND91/macIDR).

To validate our model, we tested macIDR against the previously withheld test set, which included a newly-generated RNA-seq experiment containing all included activation states (E-MTAB-7572). Classification of the test set revealed an accuracy above 0.95 with both high sensitivity (>0.98) and specificity (>0.83; Table 2). In total, 75 out of 79 test samples were correctly classified (Figure 3B). Notably, for three of the four misclassified samples, the second-best prediction was the activation model as reported by the authors. Investigation of the four misclassified samples revealed that most errors were made regarding M0: two M0 datasets were classified as M-IL10 (GSM151655) and M-IFNγ (GSM1338795), and one M-dex dataset (d54D) as M0. The fourth misclassification pertained a M-LPS_late_ (GSM464241) dataset classified as M-LPS+IFNγ (Figure 3C).

**Table 2:** Classification testing.

**Macrophage**	**TP**	**FP**	**TN**	**FN**	**TNR**	**TPR**	**Accuracy**
M0	26	1	50	2	0.98	0.93	0.96
M-LPS_early_	6	0	73	0	1.00	1.00	1.00
M-LPS_late_	6	0	72	1	1.00	0.86	0.99
M-LPS+IFNγ	7	1	71	0	0.99	1.00	0.99
M-IFNγ	8	1	70	0	0.99	1.00	0.99
M-IL4	11	0	68	0	1.00	1.00	1.00
M-IL10	6	1	72	0	0.99	1.00	0.99
M-dex	5	0	73	1	1.00	0.83	0.99

*A confusion matrix representing the classifier performance on the test set. TP, True positives; FP, False positives; TN, True negatives; FN, False negatives; TNR, True negative rate/specificity; TPR, True positive rate/sensitivity*.

Next, we wanted to test the potential limitations of macIDR by classifying macrophage differentiated with granulocyte-macrophage colony-stimulating-factor (GM-CSF) stimulated MDMs (GM-MDMs) as well as non-macrophage cell types. To this end, we classified data from four different studies (4, 23–25). We observed that the GM-MDMs were classified as their M-CSF counterparts (Figures 3D,E), whereas most non-MDMs were classified as M0 (Figure 3D, Supplementary Table 3). Notably, the monocyte-derived dendritic cells (MoDCs) were recognized as M-IL4 (Figure 3D), even after stratifying for subsequent stimulation (Figure 3F). We speculate that this might due to the differentiation method (GM-CSF + IL4) used to generate MoDCs *in vitro*.

Pathway analysis of all predictor genes revealed enrichment for inflammatory pathways, such as TNFα signaling, inflammatory response and interferon gamma signaling, underscoring the importance of inflammation regulation in macrophage activation (Supplementary Figure 2A, Supplementary Table 4). A follow-up transcription factor motif analysis on the promoters of the predictor genes showed significant enrichment for macrophage-specific transcription factors, the E26 transformation-specific PU.1 (Spi1) and SpiB (Supplementary Figure 2B).

### *In vivo* Macrophage Classification

#### Alveolar Macrophages From COPD and Smoking Individuals Display Reduced M-IFNγ Signal

**Figure 4:**
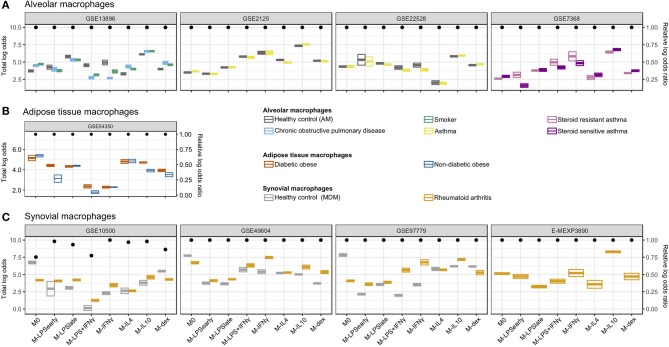
Classification of *in vivo* macrophages. Summarized classification results per dataset with cross-bars representing the mean and the standard errors of the log odds colored by the macrophage *in vivo* type. Dots above represent the log odds ratio [log(OR)] relative to the sum of the log odds ratios if all predictor genes were measured. **(A)** Alveolar macrophages obtained from smoking individuals, chronic obstructive pulmonary disease (COPD), asthma patients, as well as healthy controls. **(B)** Adipose tissue macrophages obtained from diabetic obese and non-diabetic obese patients. **(C)** Synovial macrophages obtained from rheumatoid arthritis (RA) patients and MDMs from healthy controls (HCs).

We next classified macrophages derived from patient tissues to study their semblance to *in vitro* MDMs. We investigated alveolar macrophages (AMs) obtained through bronchoalveolar lavage from smoking individuals, COPD patients, asthma patients (31), and healthy control (HCs). Overall, we found that the AMs were classified primarily as M-IL10 (Figure 4A), which appears to be driven by the *MARCO* signal. This corroborates the observation that lung tissue, specifically AMs, display significantly higher gene expression of *MARCO* relative to surrounding cells and other tissues (66, 67). Moreover, *MARCO* was found to be necessary in AMs for mounting a proper defense response (66–68). Further comparisons of the AMs derived from different patient groups, we found that the macrophage signal could be stratified according to health status where COPD- and smoker-derived AMs displayed a higher M-IL4, M-IL10, and M-dex signal and a reduced M-IFNγ and M-LPS+IFNγ signal compared to AMs obtained from HCs. This observation indicates a stronger M2- and lesser M1-like phenotype, which was also noted in previous studies (4, 31). This difference in classification signal was found to be driven by decreased log odds for C-X-C Motif Chemokine Ligand 9 (*CXCL9*) (M-IFNγ and M-LPS+IFNγ) and *CXCL5* (M-IL4) and increased log odds for *TNFAIP6* (M-IL10), and *ADORA3* (M-dex; Supplementary Figure 3).

While no clear differences were observed when comparing AMs from asthma patients to HCs, AMs from steroid-resistant asthma patients displayed a decreased M-dex signal and increased M-LPS_early_, M-IFNγ, and M-LPS+IFNγ signal relative to steroid sensitive asthma AMs (Figure 4A). The apparent difference in steroid sensitivity appeared to be caused by a difference in *TNF* and *CXCL9* signals contributing to the M-LPS_early_ and M-IFNγ classification, respectively (Supplementary Figure 3). This observation corroborates previous studies where IFNγ signaling was found to suppress glucocorticoid-triggered transcriptional remodeling in macrophages leading to the macrophage-dependent steroid-resistance (69), thereby reflecting a higher level of IFNγ in steroid-resistant relative to steroid-sensitive asthma patients (70).

#### Adipose Macrophages Show Similarity With M0 and M-IL4

We subsequently investigated visceral adipose tissue macrophages (ATMs) derived from diabetic and non-diabetic obese patients (35). Classification analysis suggested visceral ATMs showed most similarity with M0 followed by M-IL4 (Figure 4B). While we were not capable of defining a set of genes responsible for the M0 classification, we observed that the concordant expression of Monoamine oxidase A (*MAOA*) and *CCL18* contributed the most to the M-IL4 signal (Supplementary Figure 4). *MAOA* encodes a norepinephrine degradation enzyme and is expressed primarily in sympathetic neuron-associated macrophages isolated from the subcutaneous adipose tissue. In these cells, MAOA's norepinephrine clearance activity has been linked to obesity (71). Interestingly, when comparing ATMs from diabetic obese with non-diabetic obese patients, we observed a stronger signal of M-IL10 and M-LPS_early_ driven by *CCL18* and *TNF*, respectively (Supplementary Figure 4). Notably, *CCL18* expression in both visceral and subcutaneous adipose tissue has been associated with insulin-resistant obesity (72, 73). Furthermore, several studies have demonstrated that ATMs could be divided into CD11C^+^CD206^+^ and CD11C^−^CD206^+^ subpopulations (74, 75). Specifically, an increased density of the IL10 and TNFα-secreting CD11C^+^CD206^+^ ATMs in adipose tissue was associated with insulin resistance (74), which coincides with the observed increase of M-IL10 and M-LPS_early_ signals while the M-IL4 signal remained unaltered.

#### Synovial Macrophages From Rheumatoid Arthritis Patients Similar to M- IFNγ and M-IL10

Finally, we analyzed synovial macrophages (SMs) from RA patients and MDMs from HCs (26–29). Whereas, unstimulated MDMs were successfully classified as M0, SMs from RA patients were classified as either M-IL10 or M-IFNγ (Figure 4C). Specifically, RA-derived synovial macrophages (RA-SMs) from 3 studies (26, 28, 29) were classified as M-IL10, whereas samples from one study (27) were classified as M-IFNγ. Comparison of the classification signal of the RA-SMs with the HC MDMs displayed a higher signal for M-IFNγ and M-IL10 (Figure 4C). Concordantly, a previous study reported an increased gene expression of IFNγ and IL10 in RA synovial fluid mononuclear cells compared with PBMCs from both RA patients and HCs (76). Further investigation of the M-IFNγ and M-IL10 classification revealed dominant signals for M-IFNγ predictor gene *CXCL9* and for M-IL10 predictor gene *MARCO* (Supplementary Figure 5). This observation agrees with previous studies where elevated gene and protein expression of *CXCL9* was found in the synovium of RA patients compared with that of osteoarthritis patients (77, 78). Similarly, an increased presence of *MARCO* was detected in the inflamed joints, particularly in RA patients (79).

**Figure 5:**
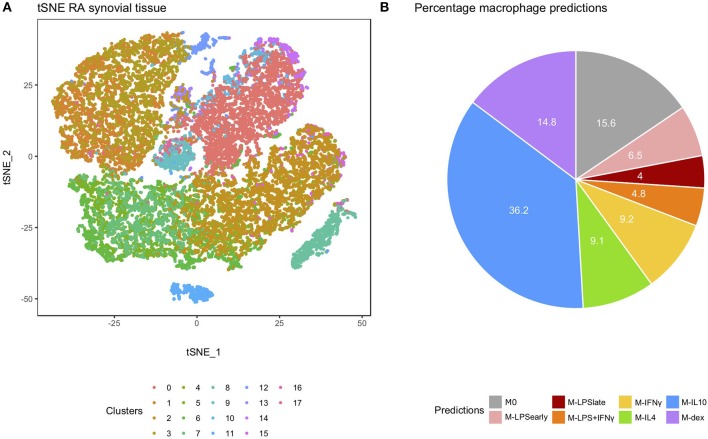
Analysis of the rheumatoid arthritis-derived synovial cells. **(A)** t-distributed stochastic neighbor embedding (tSNE) visualization of the synovial biopsy-derived cells as obtained through Louvain clustering. **(B)** Pie chart depicting the frequency of each macrophage activation model as predicted by macIDR.

We extended our analysis to single-cell RNA-seq (scRNA-seq) data from RA-derived biopsies (30). We extracted the macrophage cluster (cluster 0) based on the expression of the typical macrophage markers Spi-1 proto-oncogene (*SPI1*), complement C1q B chain (*C1QB*), CD14 molecule (*CD14*), CD68 molecule (*CD68*), and colony stimulating factor 1 receptor (*CSF1R*) (Figure 5A and Supplementary Figure 6). Classification of the individual macrophages using macIDR indicated that most macrophages were classified as M-IL10 (Figure 5B). Notably, the macrophage predictions did not match with according to RA-SM sub-clusters (Supplementary Figure 7).

## Discussion

In this study, we characterized macrophages by integrating public gene expression datasets of eight *in vitro* macrophage models. We identified both well-known and novel markers for activated macrophages across different studies. We subsequently built a classification model capable of defining and discriminating macrophage activation states and made this available as an R package called macIDR. By applying macIDR to *in vivo* macrophages, we projected the latter onto the *in vitro* macrophage model system providing insights in how disease and tissue of origin affect activation composition.

Previous macrophage characterization studies focused primarily on gathering large cohorts. Xue et al. adopted an inclusive strategy by categorizing genes into different activation states through self-organizing maps and correlation analyses (4). Instead, we sought to find consensus from published data by implementing a descriptive and an exclusive strategy represented by the meta-analysis and the elastic net classification analysis, respectively. Where the meta-analysis identified genes that were consistently differentially expressed across studies when comparing stimulated with unstimulated MDMs, elastic net classification analysis represented a rigorous feature selection approach that yielded predictor genes capable of classifying the MDMs with high accuracy. As a meta-analysis-based computational model, the strength of our approach would continuously increase as public repositories expand. Furthermore, we implemented an additional layer of robustness by performing repeated cross-validation to ensure that the final output of the elastic net regression was stable.

Many of the observed cDEGs and predictor genes have been recognized as *bona fide* markers for different activation states, such as *TNF* (M-LPS_early_), *IDO1* (M-LPS+IFNγ), *CXCL9* (M-IFNγ), and *ADORA3* (M-dex) (5) (Supplementary Table 1 and Figure 3A). We also identified several novel marker genes, which have been reported to be differentially expressed in diseases correlated with the stimuli. For example, deficiency of *NOV* (also known as *CCN3*), a down-regulated cDEG when comparing M-LPS_late_ and M-IFNγ with M0, increases foam cell formation and exacerbates atherosclerosis in mice (80, 81). Similarly, the regulation of *NOV* might play a bridging role in inflammatory response and lipid accumulation in macrophages during atherogenesis as both toll-like receptor 4 and IFNγ-signaling represent major regulators in atherosclerosis (82). Pathway and transcription factor motif analyses of the predictor genes revealed enrichment for inflammatory pathways and macrophage transcription factors PU.1 and SpiB. As both key transcription factors drive macrophage differentiation (83), our observation suggests that macrophage activation is determined by the regulation of the lineage-specific inflammation. We also observed that some predictor genes were associated to pathways not typically described within the context of macrophage activation. For example, ribosomal proteins (RPL) 6 and 9 were found to be negative predictor genes of M-LPS_early_ and are typically involved in translation. This finding might be akin to the downregulation of ribosomal translation during the maturation of MoDCs with LPS (84). Another example pertains M-IL4 for which we observed changes in serotonin processing. We observed that serotonin receptor 5-Hydroxytryptamine Receptor 2B (*HTR2B*) (76) was represented by a negative log odds ratio, whereas serotonin degrading enzyme *MAOA* (85) was represented by a positive log odds ratio. Together, it appears as though M-IL4 favors the absence of serotonin, despite the fact that serotonin reportedly attenuates the secretion of the pro-inflammatory cytokine TNFα (86). While it remains enticing to describe the functionality of the novel macrophage genes based on previous literature, it should be understood that the current study is associative in nature with the reported genes being different in expression. Our observations warrant future mechanistic studies that elucidate the physiological and functional relevance of these genes within macrophages.

To test the limits of the classification model, we classified non-MDM cells. Classification of MoDCs indicated that they were most similar to M-IL4, even after treatment with pro-inflammatory agents such as LPS. This observation is likely due to the method used to generate MoDCs, where monocytes are differentiated with GM-CSF and IL4. Notably, while the MoDCs were classified primarily as M-IL4, some MoDCs matured with LPS for 24 h displayed a mildly increased signal toward M-LPS+IFNγ and M-LPS_late_. Similarly, immature MoDCs (DC0) displayed a slightly higher response for M0. All other non-macrophage cells were classified as M0. While the log odds of proper M0 classifications were slightly higher than improper M0 classifications, we acknowledge that no clear threshold could be defined. We are unsure why M0 was predicted as a class for non-MDM and non-MoDC datasets, but we speculate that it might be related to the M0 class being represented by most predictor genes relative to the other classes. We therefore recommend potential users of macIDR to determine *a priori* that their dataset of interest represents macrophages either experimentally or using *in silico* cell-composition estimation methods, such as CIBERSORT (87) or xCell (88).

As macIDR was capable of properly classifying differentially differentiated *in vitro* MDMs, we applied it to *in vivo* macrophages with the goal of extracting activation information. We observed differences in predictions, such as reduced signals of M-IFNγ and M-LPS+IFNγ and increased signals of M-IL4, M-IL10, and M-dex when comparing AMs from COPD patients with HCs. Moreover, we observed that the tissue of origin had a large impact on the macrophage classification with *in vivo* tissue-resident macrophages obtained from HCs not always being classified as M0 as was observed for the AMs. Since some *in vivo* macrophages obtained from healthy tissue were classified as *in vitro* activated MDMs, unstimulated *in vitro* MDMs (M0) likely do not reflect the basal state of all tissue-resident macrophages underpinning the importance of how multiple factors in the microenvironment shape the transcriptome. Our results suggest that for some *in vitro* models, using activated MDMs might achieve a more comparable phenotype to the *in vivo* tissue macrophages that express the tissue transcriptomic signatures.

Unlike the AMs, no transcriptomic data was available of SMs from healthy donors. We were therefore unable to conclude whether the M-IL10 and M-IFNγ predictions for the RA samples were tissue-specific or disease-associated. Though samples from different studies were classified as M-IL10 or M-IFNγ, these two activation states appeared to be the highest two predicted classes among all recruited datasets. Characterization studies on RA-SMs suggested that they represent multiple subpopulations, such as the CD163^+^ anti-inflammatory tissue-resident macrophages and the S100A8/9^+^ pro-inflammatory macrophages recruited from peripheral monocytes (89). As *in vivo* macrophages represent a more heterogeneous population, prediction of mixed cell clusters using macIDR will return signals from multiple different models. The classification could therefore reflect perturbations of the macrophage composition associated with disease states. Since scRNA-seq enables deconvolution of cellular identities, we reasoned that integrating macIDR with single-cell profiling would enhance the understanding of *in vivo* macrophage heterogeneity in diseases and improve the resolution of cellular clusters as classified functional groups. By applying macIDR to the macrophages observed in the RA synovial samples obtained through scRNA-seq we found that most macrophages were M-IL10-like. However, the predictions did not match with the observed macrophage sub clusters suggesting that while RA-SMs are composed of different macrophage subsets, they might not necessarily coincide with the eight *in vitro* models.

In conclusion, our work represents a practical and robust platform-independent approach to characterize and classify *in vitro* macrophages. We anticipate that our approach could be expanded to multiple cell types and states, not only for modeling the activation, but also for characterizing the functional cell groups *in vivo*. Altogether, we have established a consensus gene expression profile and built a classification model called macIDR that is capable of deconvoluting heterogeneous signals both *in vitro* and *in vivo*.

## Data Availability Statement

The processed RNA-seq counts are available in the ArrayExpress repository E-MTAB-7572. The raw RNA-seq reads are available in the European Genome-phenome Archive under the accession number: EGAS00001003451 to protect the privacy of the individuals in accordance to Dutch privacy laws. All public datasets analyzed during the current study are available in the Gene Expression Omnibus, ArrayExpress, and dbGaP repositories. Studies from the GEO repository include: GSE18686, GSE19765, GSE30177, GSE47538, GSE5099, GSE57614, GSE61880, GSE79077, GSE85346, GSE99056, GSE100382, GSE55536, GSE80727, GSE82227, GSE99056, GSE75938, GSE46903, GSE93776, GSE10500, GSE49604, GSE97779, and GSE13896. Studies from the ArrayExpress repository include: E-MEXP-2032, E-MTAB-3309, E-MTAB-5095, E-MTAB-5913, and E-MEXP-3890. The macIDR package is stored in the GitHub repository at https://github.com/ND91/macIDR. The study from the dbGaP repository pertained phs001529.v1.p1. The scripts for performing the analyses are stored in the GitHub repository at https://github.com/ND91/PRJ0000004_MACMETA.

## Author Contributions

H-JC performed the literature search. H-JC and GG cultured the cells, isolated the RNA for the RNA sequencing analysis. AL downloaded, cleaned and performed the *in silico* analyses of the public and in-house generated data. H-JC, AL, PH, and MW decided on the inclusion criteria for the public data. H-JC and AL drafted the manuscript. PH and MW conceived the study and participated together with EF, WJ, and MM in the design and coordination of the study. All authors read and approved the final manuscript.

### Conflict of Interest

GlaxoSmithKline provided support in the form of salary for author AL. GlaxoSmithKline and Novartis provided support in the form of salary for author EF. GlaxoSmithKline and Novartis had no additional role in the study design, data collection and analysis, decision to publish, or preparation of the manuscript. Funding for open access charge will be covered by Amsterdam University Medical Centers, University of Amsterdam. The remaining authors declare that the research was conducted in the absence of any commercial or financial relationships that could be construed as a potential conflict of interest.
