# Supplementary figures and images for: Meta-Analysis of in vitro-Differentiated Macrophages Identifies Transcriptomic Signatures That Classify Disease Macrophages in vivo
Source: Front Immunol. 2019 Dec 11;10:2887. doi: 10.3389/fimmu.2019.02887 (PMC6917623; doi:10.3389/fimmu.2019.02887)

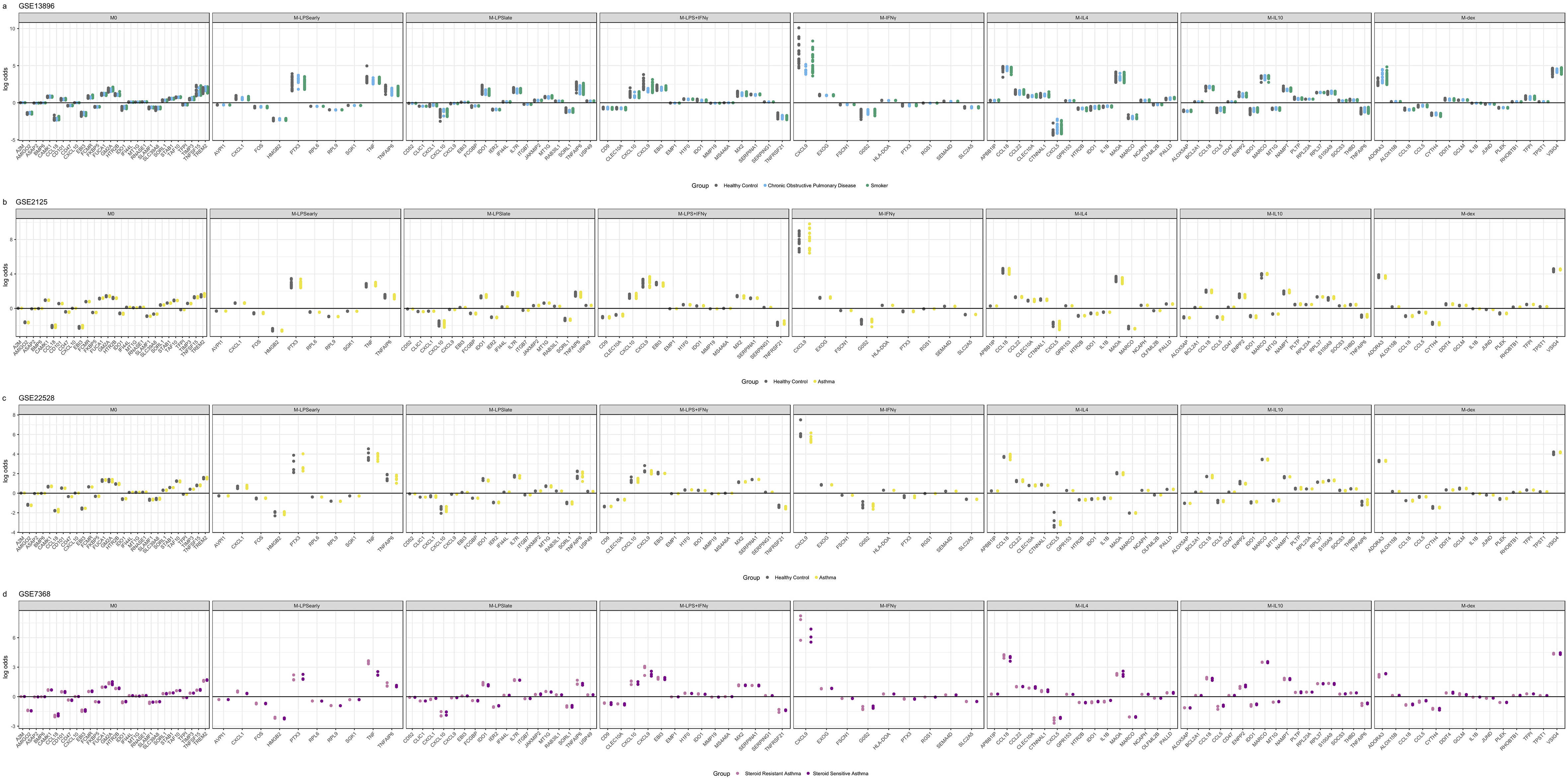

Supplement: Supplementary file 3 [file Image_3.TIFF]

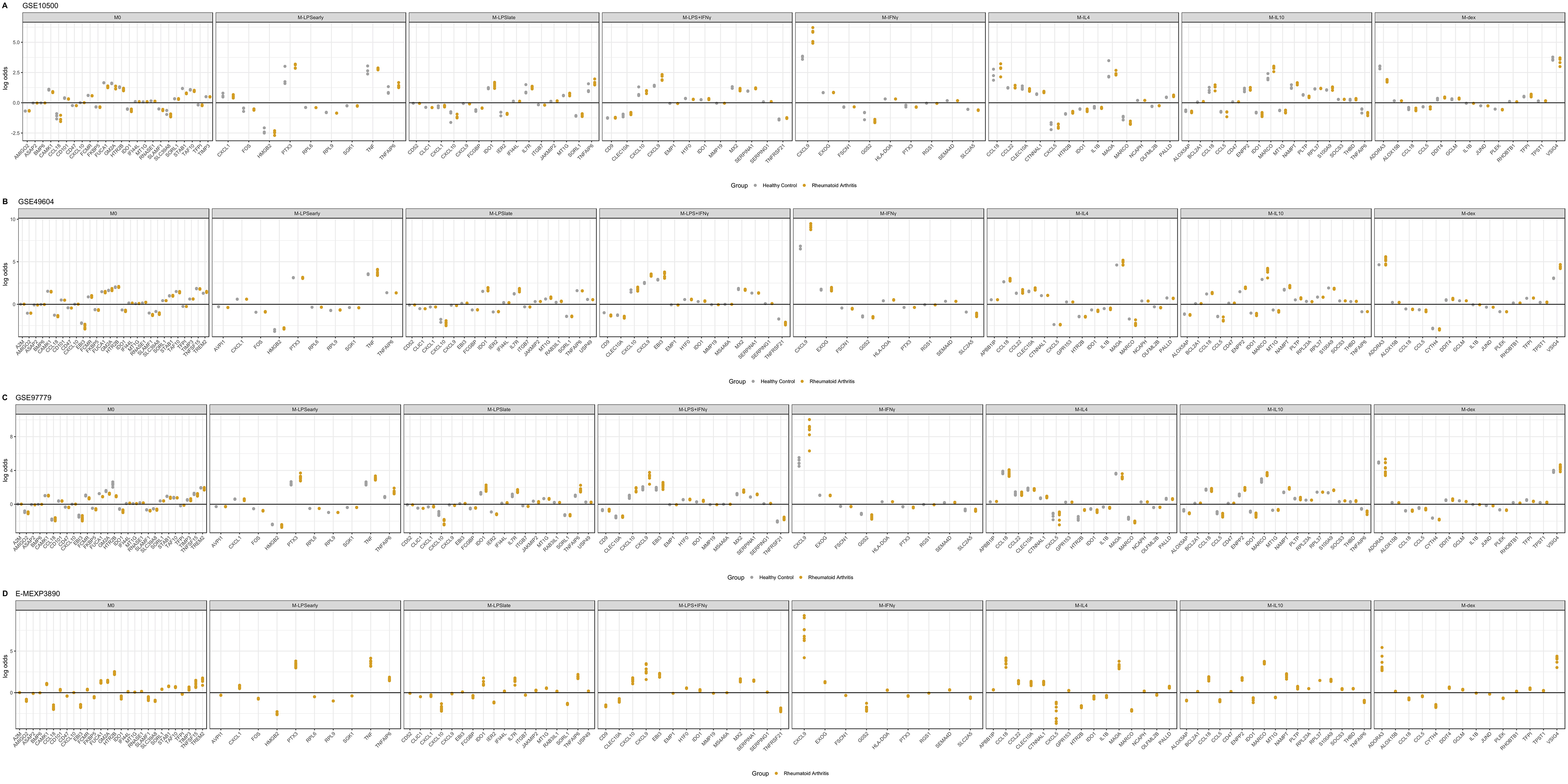

Supplement: Supplementary file 5 [file Image_5.TIFF]
