# Supplementary material for: Meta-Analysis of in vitro-Differentiated Macrophages Identifies Transcriptomic Signatures That Classify Disease Macrophages in vivo
Source: Front Immunol. 2019 Dec 11;10:2887. doi: 10.3389/fimmu.2019.02887 (PMC6917623; doi:10.3389/fimmu.2019.02887)
Supplement: Supplementary file 12 [file Data_Sheet_1.pdf]

Supplementary Material

1 Supplementary Figures and Tables

1.1 Supplementary Figures

Supplementary Figure 1. Plot representing the log odds ratio distribution as obtained from the 500 iterations of ten-fold cross-validation. Red and blue represent stable predictor genes with positive and negative coefficients respectively. Grey represents unstable predictor genes.

Supplementary Figure 2. (A) Pathway overrepresentation analysis of the predictor genes ranked by *p*-value. **(B)** Motif overrepresentation analysis of the predictor genes ranked by *p*-value. Columns represent the consensus sequence, the motif name, the *p*-value, the BH-adjusted *p*-value (q-value), the percentage of provided genes with the motif, and the percentage genes in the background with the motif.

Supplementary Figure 3. Log odds per gene for alveolar macrophage classification. Plots of the log odds per gene for the studies facetted by the different macrophage class for studies: **(A)** GSE13896, **(B)** GSE2125, **(C)** GSE22528, and **(D)** GSE7368.

Supplementary Figure 4. Log odds per gene for adipose tissue macrophage classification. Plots of the log odds per gene for the studies facetted by the different macrophage class for study: GSE54350.

Supplementary Figure 5. Log odds per gene for the synovial macrophage classification. Plots of the log odds per gene for the studies facetted by the different macrophage class for studies: **(A)** GSE10500, **(B)** GSE49604, **(C)** GSE97779, and **(D)** E-MEXP-3890.

Supplementary Figure 6. Visualization of the single-cell RNA-seq data using t-distributed stochastic neighbor embedding (tSNE) colored for the macrophage markers SPI1, C1QB, CD14, CD68, and CSF1R.

Supplementary Figure 7. Visualization of the single-cell macrophages using t-distributed stochastic neighbor embedding (tSNE) colored for the predicted macrophage class. The plotting character represents the cluster identified through Louvain clustering.

1.2 Supplementary Tables

Supplementary Table 1. Table containing results of the meta-analysis. Separate tabs contain the results for the different comparisons of the meta-analysis. Columns “Entrez” and “Gene” represent the Entrez gene ID and the HGNC symbol respectively. The column “mu” represents the unbiased estimator of the effect size and the “mu_var” represents the unbiased estimator of the variance. The columns “Z”, “Z_pval”, and “Z_pvalBH” represent the Z-statistic, the associated p-value and the Benjamini-Hochberg adjusted p-value.

Supplementary Table 2. A table containing the results of the enriched canonical pathways for each comparison made for the meta-analysis.

Supplementary Table 3. Log odds for the verification datasets. An Excel file where each tab represents the classification signal as depicted in log odds for each individual study. Each tab contains a table representing the log odds separated by predictor genes to depict the contribution of each gene to the classification. Each row represents a predictor gene for a particular class and each column represents a sample. Missing values for particular rows indicate that these genes were not present in the study. The last three columns represent the Entrez gene ID, HGNC gene symbol and the class to which the weights belong.

Supplementary Table 4. A table containing the aggregated results of the enriched canonical pathways from IPA (Tab 1) and pathway analysis from metascape (Tab 2) for all predictor genes.
